# The Seven Ages of Man

**Published:** 1888-11-10

**Authors:** 


### The Doctor's Pulpit.

### THE SEVEN AGES OF MAN.

### VI.—"The Lean and Slippered Pantaloon."

"The sixth age shifts
Into the lean and slipper'd pantaloon;
With spectacles on nose, and pouch on side;
His youthful hose well saved, a world too wide
For his shrunk shank; and his big manly voice,
Turning again toward childish treble, pipes
And whistles in his sound."

It has been our part to protest in detail against some of Shakespeare's points in his description of the progressive stages of man's life. This perhaps is the most suitable place for protesting against his fundamental position. All the world is *not* a stage; and all the men and women are *not* merely players. It is not necessary to assume that Shakespeare himself looked upon life as a stage-play. It is quite obvious that it may be compared with such a play; and that the analogy may be worked out in some detail is clearly shown by what Shakespeare has done in his seven ages. Further, the comparison illustrates and expresses a certain amount of truth, as is convincingly shown by the universal acceptance of it as a more or less correct description of life in many of its aspects. But for scientific and practical purposes the description of human life as a stage-play is not a true statement of the facts. It does not correspond to the eternal verities. Outwardly, the semblances are in many cases such as Shakespeare describes; the inner realities he scarcely even professes to touch.

That every man has grown, is growing, or will grow old, if he lives long enough, is a most incontrovertible fact. That many people in growing old grow "lean," in every sense of that very expressive word, is also true. But that advancing age is, or ought to be, represented by such universally-decayed powers as Shakespeare has associated with it is not in accordance with the scientific intention of things. Given a healthy constitution, which is the normal order, and a life lived in accordance with sound physiology and rational morality, then that period of advancing years taken as the poet's sixth stage of manhood will bear no more resemblance to his "lean and slippered pantaloon" than the scarecrow bears to the living rook cawing with the boldness of experience and healthy old age on the edge of his twentieth nest. Growing age has its sure diminutions! That all must admit. But it has its compensations; and in the case here supposed, of a healthy constitution to begin with and a well-lived life, the compensations are sufficient to maintain a steady balance of capable and wholesome manhood. The "lean and slippered pantaloon," with all that appertains to him, may be held up to scorn as a contemptible example of everything a man at his time of life ought not to be.

How, then, came the great master so to paint the sixth age? For it is a portrait he must have drawn from the life. Let us look at it once more. "The sixth age shifts into the lean and slipper'd pantaloon; With spectacles on nose and pouch on side; His youthful hose well saved, a world too wide For his shrunk shank; and his big manly voice, Turning again towards childish treble, pipes And whistles in his sound." Poor old fellow! Poor old boy! He is like a hunter reduced to chopped hay and meal, or a bran mash; whose only duty is to take his old lady to church on Sunday mornings and to cart half-a-ton of coals once a fortnight from the nearest railway station. But if the hunter had had the brains of a man, would that have been the old age he would have prepared for himself? Most certainly not!

The man who makes life a stage-play—and many men do so—constitutes the world his audience and his master. When he has done that, he has closed up the one spring within him which keeps youthfulness alive.

To regard time and the material world as the beginning and end of existence, of purpose and of hope, is to destroy at a blow, not only immortality in the theologian's sense, but that faculty in man which keeps him vital, green and fruitful, to the very end of life. This proposition is not here advanced as a religious conviction, but as a philosophic conclusion from data furnished by physiology, observation, and experience. Shakespeare saw the outside of life. Undoubtedly he saw deeper. But it was his business as a dramatist to make striking situations and to depict obvious figures: to paint that which all men could see and recognise with their everyday eyes. He mostly painted the common types in every class; those which were familiar to all the world. How lifelike were his pictures all the world of his own day and all the world of ours knows full well. But men of the common type, the common intellect, the common purpose, constitute the large majority of mankind. They have not the broad mental capacity which enables its possessor to look far and wide over the present, the past, and the future; the clear judgment which convinces him that there are more things in heaven and earth than can be fully comprehended by any human philosophy, the firm purpose which fixes him and keeps him fixed on the immovable rocks of openminded candour, morals inspired by the best examples of all ages, and an immortal hope. The common mind in every class does not attain to this. On the contrary, it is intellectually like the wild beast or the domestic animal. It finds itself in a certain environment, which obtrudes upon its notice certain gross and obvious facts. It masters, without much trouble, a way of living more or less comfortably within its environment, and there it rests content. The wings of its imagination it does not use, and not being used they atrophy; and that spirit which was made to be an eagle, and to see the world as it may be seen from heights that approach the sun, becomes an ostrich, and sees the world from the midst of the sand in which its head is buried.

The proposition it is wished to establish is this: that the compensations which accompany and result from advancing age, when the life has been spent on the highest intellectual and moral planes, are more than sufficient to counterbalance the inevitable diminutions and contractions. Is it worth while to establish such a proposition, supposing it to be possible? Undoubtedly! A third part of the adult life may be said to belong to what Shakespeare has called the "lean and slippered pantaloon" stage. Most men begin to grow old and to look old at sixty? If the poet's description is to be made the measure and inspiration of life after that period there is a sorry prospect for many. Not only so, but the world itself is largely influenced and even governed by men who have reached this very stage. Is it the case that we consent to be ruled by men with shrunk shanks and loosely-hanging hose, whose dry and withered limbs are the fitting index of a dry and withered brain? That is often the very opposite of the truth? Men should expect to do their best work after they have arrived at years of intellectual maturity. Very few are intellectually mature at forty. From fifty to seventy, seventy-five, or even eighty, the brain of a man who has had an originally wholesome constitution should be at its ripest and clearest. The time of the passions is past, the sobering power of long responsibility has done its work, the chastening influence of an approaching day cf account is distinctly felt, and if ever a man is steadfastly true and noble it is during that period.

Old age must come. It will come to all men in one of two forms: either in the form of the "lean and slippered pantaloon" of the dramatist, or in the form of the healthy, courageous, and buoyantly hopeful man who has devoted himself and still means to devote himself to the highest and worthiest duties that fall within his sphere. In which of the two forms it shall come to any particular man depends entirely upon the man himself and his brains. For at the bottom it is a question of intellectual capacity. The man who is merely the creature of his environment, like a sheep or an elephant, is, from a rational standpoint, a fool. Every normal man is constructed with a reason and a conscience. These are normal parts of him. Their use is essential to his normal development and perfection as a man. If he lives entirely under the control and influence of his environment his old age will be that of the lean and slippered pantaloon. If on the other hand he gives to reason and conscience their full and just freedom of action, his old age will be a period of advancing intelligence, growing wholesomeness of character, and deepening pleasure and hope

